# *Centella asiatica* Extract Attenuates Kidney Fibrosis Through Reducing Mesenchymal Transition and Inflammation in Ureteral Ligation Model in Mice

**DOI:** 10.3389/fphar.2021.621894

**Published:** 2021-03-17

**Authors:** Dwi Cahyani Ratna Sari, Santosa Budiharjo, Husnari Afifah, Destantry Jasmin, Orisativa Kokasih, Tiara Gitami Putri, Karina Arifiani, Wiwit Ananda Wahyu Setyaningsih, Nur Arfian

**Affiliations:** ^1^ Department of Anatomy, Faculty of Medicine, Public Health and Nursing, Universitas Gadjah Mada, Yogyakarta, Indonesia; ^2^ Undergraduate Student, Faculty of Medicine, Public Health and Nursing, Universitas Gadjah Mada, Yogyakarta, Indonesia; ^3^ Siloam Hospitals Group, Ancillary and Medical Affairs Regional Head, Depok, Indonesia

**Keywords:** *Centella asiatica*, unilateral ureteral obstruction, inflammation, mesenchymal transition, kidney fibrosis

## Abstract

**Background:** Kidney fibrosis is the common final pathway of chronic kidney disease (CKD)*,* and is characterized by inflammation, mesenchymal transition with myofibroblast formation and epithelial to mesenchymal transition (EMT). *Centella asiatia* (CeA) is an herb that has a reno-protective effect. However, its mechanism of action in kidney fibrosis has not been elucidated.

**Aim:** To elucidate the effect of CeA in amelioration of kidney fibrosis in a unilateral ureteral obstruction (UUO) model and focus on mesenchymal transition and inflammation.

**Methods:** Unilateral ureteral obstruction was performed in male Swiss-background mice (age: 2–3 months, weight: 30–40 g, UUO group *n* = 6) to induce kidney fibrosis. Two doses of CeA extract with oral administration, 210 and 840 mg/kg body weight were added in UUO (U+C210 and U+C840 groups, each *n* = 6). The sham operation procedure was performed for the control group (SO, *n* = 6). The mice were euthanized at day-14 after operation. Tubular injury and interstitial fibrosis area fractions in kidney tissues of the mice were quantified based on periodic acid-Schiff (PAS) and Sirius Red (SR) staining. Immunostaining was performed for examination of fibroblast (PDGFR-β), myofibroblast (α-SMA), Monocyte Chemoattractant Protein-1 (MCP-1) and macrophage (CD68), meanwhile double immunofluorescence was performed with PDGFR-β and α-SMA. Reverse transcriptase-polymerase chain reaction (RT-PCR) was performed to examine mRNA expression of TGF-β, Collagen-1, Snail, E-cadherin, vimentin, fibroblast-specific protein 1 (FSP-1), CD68, toll-like receptor 4 (TLR4), and MCP-1.

**Results:** We observed a significantly higher interstitial fibrosis area fraction and tubular injury (*p* < 0.001) with fibroblast expansion and myofibroblast formation in the UUO group than in the SO group. These findings were associated with higher mRNA expression of TGF-β, Collagen-1, Snail, vimentin, FSP-1, CD68, TLR4, and MCP-1 and lower mRNA expression of E-cadherin. The U+C840 group had a significantly lower tubular injury score and interstitial fibrosis area fraction, which associated with downregulation of mRNA expression of TGF-β, Collagen-1, Snail, vimentin, FSP-1, CD68, TLR4, and MCP-1, with upregulation of mRNA expression of E-cadherin. Immunostaining observation revealed the U+C840 group demonstrated reduction of macrophage infiltration and myofibroblast expansion.

**Conclusion:** CeA treatment with dose-dependently ameliorates mesenchymal transition and inflammation in kidney fibrosis in mice.

## Introduction

Chronic kidney disease (CKD) is a global public health problem, and its incidence is increasing concomitantly with the aging population, even in Indonesia. The World Health Organization estimated approximately 58 million deaths due to kidney diseases and around 35 million deaths were attributed to CKD (Neuhofer and Pittrow, 2006; Levey et al., 2007). A survey conducted by the US Centers for Disease Control and Prevention reported an increase in the prevalence of CKD from 12% in 1988–1994 to 15% in 2003–2006, especially in people aged 60 years or above. Meanwhile, a survey conducted by Indonesia Nephrology Association reported that approximately 12.5% of the population or 25 million people in Indonesia have decreased renal function. Adults with diabetes, hypertension, or both, have a higher risk of developing CKD than those without these conditions. Other risk factors leading to CKD are cardiovascular disease, obesity, and a familial history of CKD (Centers for Disease Control and Prevention, 2014). Globally, diabetes mellitus is known as a major cause of CKD. In the last decade in Indonesia, diabetic nephropathy was considered the main cause of CKD, although in recent years, the cause has gradually shifted to hypertension (PERNEFRI, 2015).

Fibrosis is defined as a wound healing response that goes out of control, and there is abundant accumulation of extracellular matrix (ECM) in the affected tissue. Kidney fibrosis might cause loss of normal renal function due to the replacement of normal tissues with fibrosis scars (Wynn, 2007), as the hallmark of progressive renal diseases (Darby et al., 2014). Some studies have reported tubulointerstitial involvement in the deterioration of renal function, more than its glomerular counterpart. Renal tubular fibrosis is characterized by the accumulation of ECM, which consists of type I, II, III, and IV collagen; proteoglycans; and fibronectin (Darby et al., 2014). Myofibroblast roles are principle ECM producing cells (Darby et al., 2014), which derive from fibroblast and pericyte through mesenchymal/myofibroblast transition (Humphreys, 2018). Interstitial fibroblasts are recognized as primary matrix-producing cells as the sources of the ECM (Strutz and Zeisberg, 2006). The damage of tubulointerstitial due to fibrosis and tubular injury becomes a good predictive value of CKD due to its reverse correlation with deterioration of renal function (Duffield and Humphreys, 2012), furthermore myofibroblast formation with *de novo* α-smooth muscle actin (α-SMA) synthesis is strongly associated with kidney diseases and fibrosis progression (Djudjaj and Boor, 2018).

Unilateral ureteral obstruction (UUO) is an experimental model that is used to generate progressive renal fibrosis. Renal fibrosis is a hallmark of progressive renal diseases caused by various etiologies and is characterized by inflammation, transformation of myofibroblasts, and ischemic conditions (Ishidoya et al., 1995). The UUO model is useful to present the progression of renal disease biomarkers, which require intervention before there is an irreversible renal injury, as well as the effect of new therapies. The use of the UUO model permits manipulation of timing, severity, and duration of the obstruction. A time period of 1–2 weeks is needed to induce severe hydronephrosis in mice using this model (Chevalier et al., 2009). UUO might cause hemodynamic changes in the kidney and induce macrophage infiltration, leading to tubulointerstitial fibrosis. Various cytokines such as tumor necrosis factor α (TNF-α), interleukin 6 (IL-6), IL-1, transforming growth factor β (TGF-β), monocyte chemoattractant protein 1 (MCP-1), IL-8, regulated on activation, normal T cell expressed and secreted protein, toll-like receptor 4 (TLR4), cluster of differentiation 68 (CD68), and other proinflammatory cytokines are known to contribute to CKD (Devarajan, 2006).

The UUO technique can simulate renal failure (Klahr et al., 1988), By causing a progressive renal fibrosis, and is associated with the mesenchymal transition of epithelial cells through epithelial–mesenchymal transition (EMT) in renal tubules and ECM synthesis (Sato et al., 2003). EMT is defined as the intersection of inflammatory conditions with progressive renal fibrosis and cancer. Cellular characteristics of EMT include loss of epithelial cell polarity, transition of cuboid cells to fibroblastic cells, decrease of epithelial cell markers, and enhancement of mesenchymal markers (Kalluri and Neilson, 2003). EMT might enhance expression of some transcription factors, including heat shock protein 47, collagen 1 (α1), collagen 2 (α2), N-cadherin, and vimentin and also increases cytoskeletal protein synthesis (Robson et al., 2006). Further, EMT may lead to loss of epithelial cell markers such as E-cadherin, syndecan-1, and zonula occludens-1 (ZO-1). Tubular injury promotes EMT in hyperuricemia models, as demonstrated by the upregulation of vimentin and downregulation of E-cadherin (Setyaningsih et al., 2018).

*Centella asiatica* or CeA is a medicinal herb that has been used in the eastern part of the world for a long time and has recently become popular in the West. The herb has also been used by the Javanese and people from other regions of Indonesia. Since the 19th century, CeA has been used in the treatment of various skin conditions such as leprosy, eczema, psoriasis, and ulcers. It has also been used to treat other medical conditions such as diarrhea, fever, and genitourinary tract infections. This herb belongs to the *Umbelliferae (Apiaceae)* family and can be grown in tropical and subtropical countries, especially in humid places. Most parts of CeA are useful for medication, including flowers, leaves, stems, and roots. The primary active components of CeA are triterpenoid saponins, which might have effects on the process of wound healing through inhibition of collagen production at the wound site. Other components of CeA, such as centellosides and its derivatives, are found to be effective in treating venous hypertension. Topical use of CeA might be effective in wound healing, by enhancing the production of type I collagen and decreasing inflammatory responses and myofibroblast production (Gohil et al., 2019). However, the application of CeA in the treatment of kidney fibrosis has not been elucidated yet, especially in relation with mesenchymal transition and inflammation. Therefore, in this study, we investigated the effect of CeA treatment on kidney fibrosis.

## Materials and Methods

### Animal Experiment

This study was approved by the Medical and Health Research Ethics Committee of the Faculty of Medicine, Public Health and Nursing, Universitas Gadjah Mada, Yogyakarta, Indonesia, based on a statement letter of ethical expedience (KE/FK/0300/EC/2017). Male Swiss mice (*n* = 24, age: 2–3 months, weight: 30–40 g) were obtained from the Experimental Animal Care Unit (UPHP) LPPT of Universitas Gadjah Mada. These mice were randomly divided into four groups: sham operation group (SO, *n* = 6), UUO group (*n* = 6), UUO group treated orally with CeA extract at a dose of 210 mg/kg body weight (U+C210 group, *n* = 6), and UUO group treated orally with CeA extract at a dose of 840 mg/kg body weight (U+C840 group, *n* = 6). The mice were housed in a 50 × 30 × 15 cm plastic cage according to their grouping, with a maximum of three mice per cage, for 14 days. The cage environment was maintained under a 12:12 h natural light:dark cycle at 21°C and a humidity level of 40–60%. The mice were provided with standard American Institute of Nutrition Rodent Diet AIN 93A food and water *ad libitum*.

UUO was performed on the mice of three groups (UUO, U+C210, and U+C840 groups) to induce kidney fibrosis. Briefly, the mice were anesthetized by intraperitoneal administration of sodium pentobarbital (10 mg/kg body weight). The left flank region was dissected, and the left ureter was visualized and ligated at two sites. The abdomen was closed with sutures. The sham operation procedure was performed for the control group (SO).

### Kidney Harvesting

The mice were anesthetized with intraperitoneal administration of sodium pentobarbital (10 mg/kg body weight), and the abdomen and thorax were dissected to visualize the heart and kidney. The organs were perfused with 0.9% NaCl from the left ventricle using a Perista pump (Atto^®^; Catalog. No. SJ-1211H). The left kidney was harvested, and half of it was kept in RNA*later*™ (Ambion, Cat. No. AM7021) stabilization solution for RNA and protein extraction, and the other half was fixed in 4% paraformaldehyde (PFA) in phosphate-buffered saline (PBS) for paraffin processing of the tissue.

### Fibrosis Area Fraction and Tubular Injury Score Quantification

Paraffin sections of 4-μm thickness were deparaffinized and stained with Sirius Red (SR) to quantify the fraction area of interstitial fibrosis. Images of 15 randomly selected fields were captured using OptiLab software (Olympus, Cat. No. CX22) at ×400 magnification. The tubular injury score was assessed on the base of histopathology of the tubules and was graded from 0 to 4 (0: no change; 1: changes affecting <25% of the section; 2: changes affecting 25–50% of the section; 3: changes affecting 51–75% of the section; and 4: changes affecting >75% of the section). The tubular injury score was also assigned according to the tubulointerstitial lesions (tubular atrophy, tubular dilatation, loss of brush border, intraluminal casts, interstitial inflammation, and fibrosis). The interstitial fibrosis area fraction was quantified using ImageJ software.

### Western Blot Analysis

Kidney protein was extracted from the tissues according to the manufacturer’s protocol, using Pro-Prep™ (Intron Biotechnology, Cat. No. 17081). Fifty milligrams of the kidney tissue were homogenized with 600 μl of Pro-Prep™ solution and centrifuged at 15,000 rpm at 4°C for 20 min. The supernatants were stored at −80°C until they were assayed. A total of 40 g of protein was separated using 10% sodium dodecyl sulphate–polyacrylamide gel electrophoresis (SDS-PAGE) and transferred to a polyvinylidene fluoride membrane (PVDF). Then, the membrane was incubated with mouse monoclonal α-smooth muscle antibody (α-SMA) (Sigma, Cat. No. A2547, 1:500 dilution) and rabbit polyclonal β-actin antibody (Abcam, Cat. No. ab8227, 1:1,000 dilution). Next, the membrane was incubated with blocking serum and horseradish peroxidase (HRP)-labeled secondary antibody. Finally, the proteins were visualized using ECL Prime Western Blotting Detection Reagents (GE Healthcare, Cat. No. RPN2232). The blots were photographed with a Geldoc machine (Geldoc Syngene Gbox Seri Chemi xrq).

### RNA Extraction

The RNA from the kidney tissue was extracted with TRIzol RNA solution (GENEzol™, Cat. No. GZR100), and the concentration was quantified with NanoDrop™. The cDNA was synthesized using ReverTra Ace™ (Toyobo^®^, Cat. No. TRT-101) with the addition of random primers (TAKARA^®^, Cat. No. 3801) and deoxynucleoside triphosphate (dNTP, TAKARA^®^, Cat. No. 4030). Reverse transcriptase-polymerase chain reaction (RT-PCR) was carried out to examine the following genes:

**Table:** 

No	Genes	Sequences		Annealing temperature (°C)
1	Snail	F	5′-CTG​CTT​CGA​GCC​ATA​GAA​CTA​AAG-3′	51
		R	5′-GAG​GGG​AAC​TAT​TGC​ATA​GTC​TGT-3′	
2	E-cadherin	F	5′-CAG​CCT​TCT​TTT​CGG​AAG​ACT-3′	58
		R	5′-GGT​AGA​CAG​CTC​CCT​ATG​ACT​G-3′	
3	Vimentin	F	5′-CGG​AAA​GTG​GAA​TCC​TTG​CA-3′	58
		R	5′-CAC​ATC​GAT​CTG​GAC​ATG​CTG-3′	
4	Fibroblast-specific protein 1 (FSP-1)	F	5′-GAT​GAG​CAA​CTT​GGA​CAG​CA-3′	53
		R	5′-ATG​TGC​GAA​GAA​GCC​AGA​GT-3′	
5	CD68	F	5′-CAT​CAG​AGC​CCG​AGT​ACA​GTC​TAC​C-3′	60
		R	5′-AAT​TCT​GCG​CCA​TGA​ATG​TCC-3′	
6	TLR4	F	5′-GGG​CCT​AAA​CCC​AGT​CTG​TTT​G-3′	57
		R	5′-GCC​CGG​TAA​GGT​CCA​TGC​TA-3′	
7	MCP-1	F	5′-CTA​CAG​ACA​ACC​ACC​TCA​AGC​ACT​TCT​GTA​G-3′	60
		R	5′-GGC​ATC​ACA​GTC​CGA​GTC​ACA​C-3′	
8	Glyceraldehyde 3-phosphate dehydrogenase (GAPDH)	F	5′-TCA​ACA​GCA​ACT​CCC​ACT​CTT​CCA-3′	57
		R	5′-ACC​CTG​TTG​CTG​TAG​CCG​TAT​TCA-3′	
9	Transforming growth factor- β (TGF-β)	F	TTC​CGC​TGC​TAC​TGC​AAG​TCA	60
		R	GGG​TAG​CGA​TCG​AGT​GTC​CA	
10	Collagen-1	F	ATG​CCG​CGA​CCT​CAA​GAT​G	60
		R	TGA​GGC​ACA​GAC​GGC​TGA​GTA	

The amount of the reagents was based on the instructions on the kit (GoTaq Master Mix, Cat. No. M7122). The PCR conditions were as follows: initial denaturation at 94°C for 2 min, 35 cycles of denaturation at 94°C for 10 s, annealing for 30 s, and extension at 72 °C for 1 min. The final extension phase ended at 72°C for 10 min.

### Immunohistochemical Staining of Macrophages and Myofibroblasts

The kidney tissue was embedded in 4-μm thick paraffin sections. Then, it was deparaffinized and rehydrated using xylene and alcohol series. The specimens were stained with SR to measure the fraction area of interstitial fibrosis and with periodic acid-Schiff (PAS) to determine tubular injury. This step was followed by antigen retrieval, blocking peroxidase using 3% H_2_O_2_ in PBS solution, and blocking non-specific antigen using Background Sniper for immunostaining. The slides were incubated with α-SMA (1:400 dilution, Sigma, Cat. No. A2547) and CD68 (1:400 dilution, Abcam, Cat. No. ab955), PDGFR-β (1:200 dilution, Abclonal, Cat. No. A2180), MCP-1 (1:100 dilution, Abcam, Cat. No. ab25124) as the primary antibodies; TrekAvidin-HRP label, conjugated to anti-rabbit Trekkie universal Link (Biocare Medical^®^), as the secondary antibody; and diaminobenzidine tetrahydrochloride (DAB). The α-SMA immunostaining was applied to measure myofibroblast expansion, and CD68 antibody was used for counting macrophage cells. The quantification was performed from 15 fields for each sample at ×400 magnification, using ImageJ software.

### Immunofluorescence of α-SMA and PDGFR-β

The paraffin section of the kidney was deparaffinized and rehydrated using xylene and alcohol in gradients. Next, the sections were heated using citrate buffer pH 6 for 20 min for antigen retrieval and followed by blocking non-specific antigen using blocking serum for 20 min. Then, the slides were incubated with primary antibodies, PDGFR-β (1:200 dilution, Abclonal, Cat. No. A19531) and α-SMA (1:400 dilution, Sigma, Cat. No. A2547), overnight. On the following day, the slides were incubated with secondary antibody, goat-anti-mouse (Abclonal, Cat, No. AS076), and goat-anti-rabbit (Abclonal, Cat. No. AS039) for 1 h, and DAPI staining for 20 min. Finally, the slides were observed under a confocal microscope (Zeiss, Cat. No. LSM900).

### Statistical Analysis

The data collected were analyzed using one-way analysis of variance (ANOVA) test, if normally distributed, and Kruskal-Wallis test, if not normally distributed. The values of *p* < 0.05 were considered statistically significant. Statistical analyses were performed using SPSS Software version 22.0 (SPSS Inc., Chicago, United States).

## Results

### CeA Extract Attenuates Interstitial Fibrosis in a Dose-Dependent Manner

**FIGURE 1:**
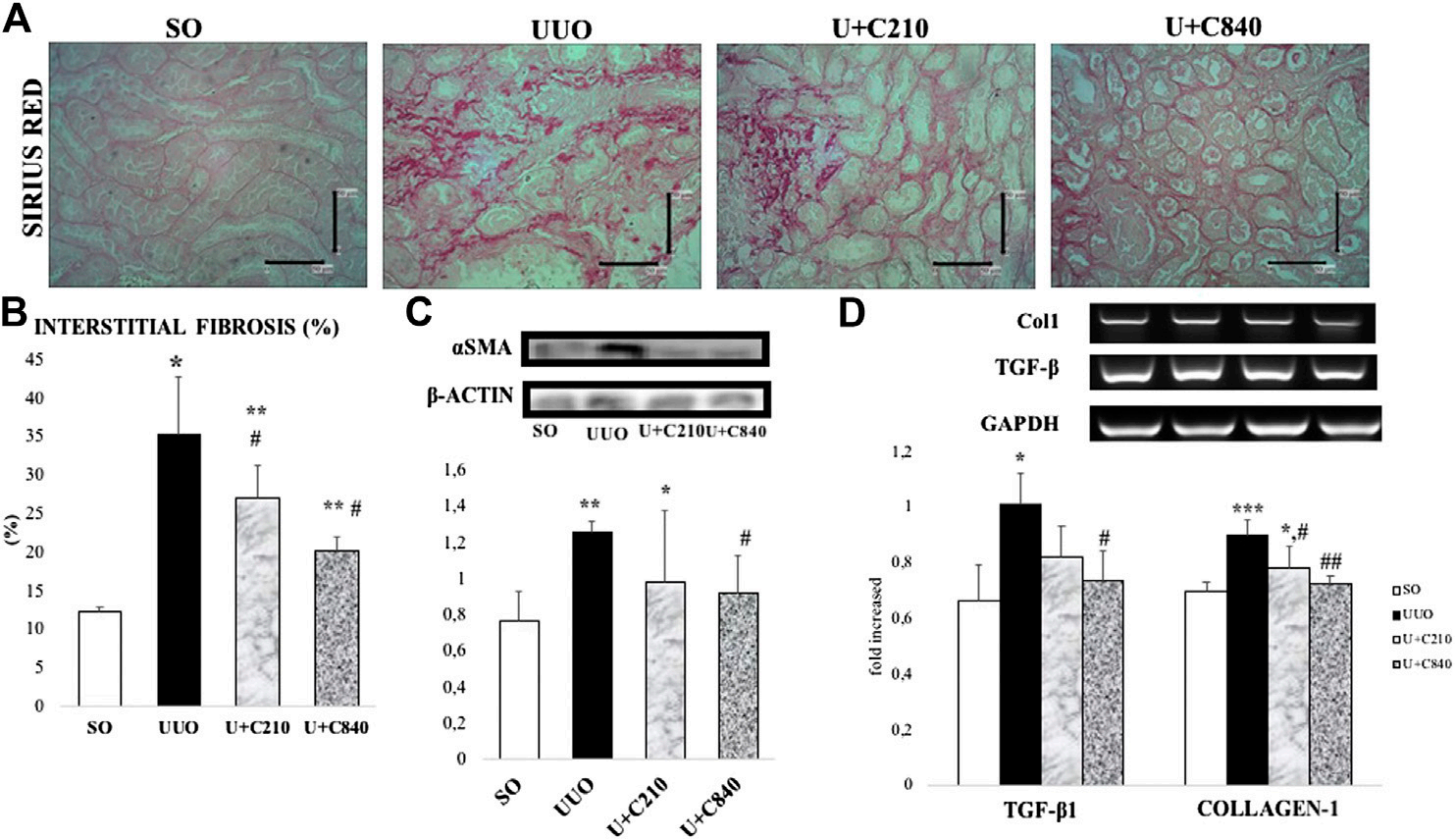
CeA treatment attenuated fibrosis. **(A)** Representative picture of tubulointerstitial fibrosis based on Sirius Red staining demonstrated red color staining of fibrosis in interstitial areas. **(B)** Quantification of interstitial fibrosis area fraction from SR staining. **(C)** Western blot analysis of aSMA protein expression. **(D)** Representative results and analysis of Collagen-1 and TGF‐β mRNA expression based on RT‐PCR. *p < 0.05 vs SO; **p < 0.001 vs SO, #p < 0.05 vs UUO; ##p < 0.01 vs UUO, ‡p < 0.05 vs U+C210, ‡‡p < 0.01 vs U+C210.

The UUO group showed an increase in collagen deposition in the tubulointerstitial area. The interstitial area fraction of fibrosis (*p* < 0.01) increased significantly in the UUO group compared with the SO group. The administration of CeA showed a dose-dependent decrease in collagen deposition. The CeA-treated group demonstrated lower interstitial fibrosis area fraction. The interstitial fibrosis decreased to greater extent in the U+C840 group compared with the U+C210 and UUO groups (Figure 1). The UUO group had significantly higher mRNA expression of not only TGF-β and Collagen-1 when compared to the SO group (*p* < 0.05) but also a significantly higher protein level of α-SMA (*p* = 0.000) when compared to the SO group. CeA treated group, especially U+C840 group had significant lower mRNA expression of TGF-β and Collagen-1, and α-SMA protein expression (*p* < 0.05) compared to UUO group (Figure 1).

### CeA Extract Attenuated Mesenchymal Transition

**FIGURE 2:**
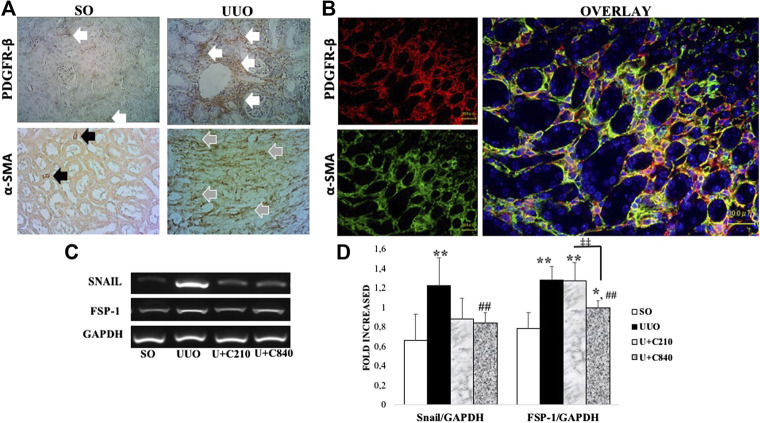
CeA treatment attenuated mesenchymal transition with downregulation of FSP-1 and snail mRNA expression. **(A)** Immunotaining of fibroblast (PDGFR-β) and myofibroblast (α-SMA). Fibroblast were stained in interstitial areas of SO group, meanwhile fibroblast expansion occurred in UUO as shown by expansion of positive staining in interstitial areas (white arrows). Positive staining of α-SMA showed smooth muscle cells of vessels (black arrows) in SO, meanwhile α-SMA revealed myofibroblast in interstitial areas of UUO group (grey arrows). **(B)** Double immunofluorescence staining demonstrated colocalization of fibroblast (red) and myofibroblast (green) which revealed fibroblast to myofibroblast transition. **(C,D)** Representative picture and semiquantitative analysis of RT-PCR analysis showed Snail and FSP-1 mRNA expressions.

We suggested that an increase in myofibroblast transition in our model. Immunostaining of fibroblast and myofibroblast markers (PDGFR-β and α-SMA) revealed postive staining in interstitial areas of UUO groups which showed mesenchymal cells expansion and myofibroblast formation (Figure 2A). Furthermore, double immunofluorescence staining demonstrated colocalization between PDGFR-β and α-SMA staining which revealed myofibroblast transition from fibroblast (Figure 2B). The transition associated with the upregulation of mRNA expression of mesenchymal cell transition markers, such as Snail and fibroblast-specific protein 1 (FSP-1), with higher expression in UUO group compared to SO group (*p* < 0.05). The CeA treatment downregulated mRNA expression of Snail (*p* = 0.000) and FSP-1 (*p* = 0.000) as shown by lower expression of snail and FSP-1 compared with the UUO group (Figures 2C,D).

### CeA Extract Reduced Tubular Injury and Epithelial–Mesenchymal Transition

**FIGURE 3:**
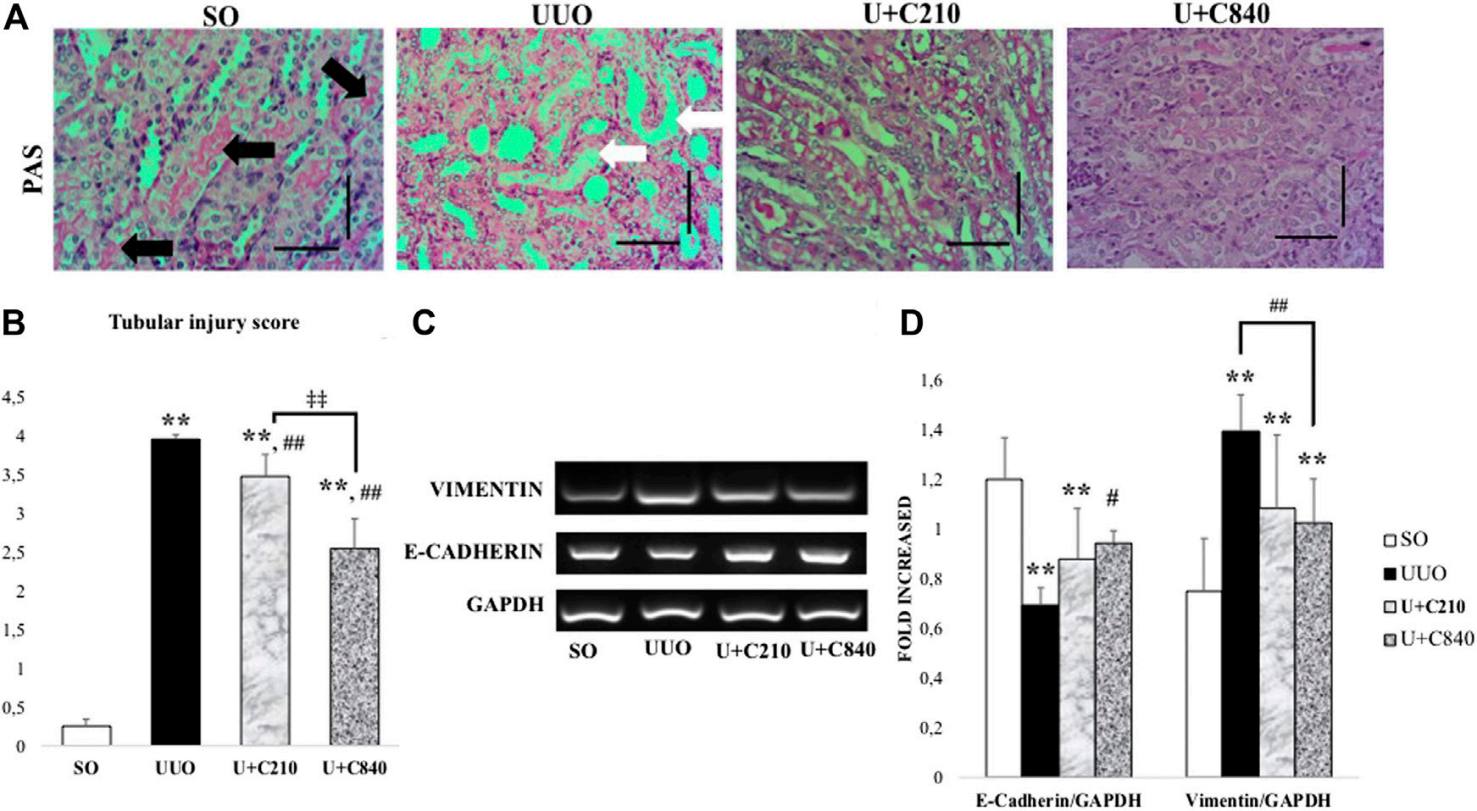
CeA treatment ameliorated tubular injury with upregulation of E-cadherin. **(A)** Representative figures of tubular injury shown by PAS staining. SO group demonstrated normal kidney architecture with brush border and intact tubules (black arrows). UUO group represented tubular injury with brush border loss, tubular dilatation and tubular epithelial cells effacement (white arrows). Ameliration of tubular injury in CeA treated group with brush border availability. **(B)** Bar charts showing tubular injury score. **(C,D)** Representative pictures and densitometry analysis of gel electrophoresis results of Vimentin and E‐Cadherin mRNA expressions based on RT‐PCR. *p < 0.05 vs SO; **p < 0.001 vs SO, #p < 0.05 vs UUO; ##p < 0.01 vs UUO, ‡p < 0.05 vs U+C210, ‡‡p < 0.01 vs U+C210.

Tubular injury occurred in the UUO group and was characterized by tubular dilatation, intraluminal cast formation, epithelial cell effacement, brush border loss, and inflammatory cell accumulation. In a semi-quantitative analysis of tubular injury, the score was significantly higher in the UUO group than in the SO group. The CeA-treated groups had lower tubular injury scores, and the U+C840 group had noticeably lower tubular injury score than those of the UUO and U+C210 groups (Figure 3).

Tubular injury is associated with morphological changes in epithelial cells, such as epithelial cell effacement and EMT. EMT might occur in UUO, as shown by the significantly lower mRNA expression of E-cadherin and higher mRNA expression of vimentin in the UUO group compared with the SO group. Meanwhile, the CeA-treated groups demonstrated higher mRNA expression of E-cadherin and lower mRNA expression of vimentin when compared to the UUO group, although only the U+C840 group showed a significant difference (Figure 3).

### CeA Extract Treatment Attenuates Inflammation

**FIGURE 4:**
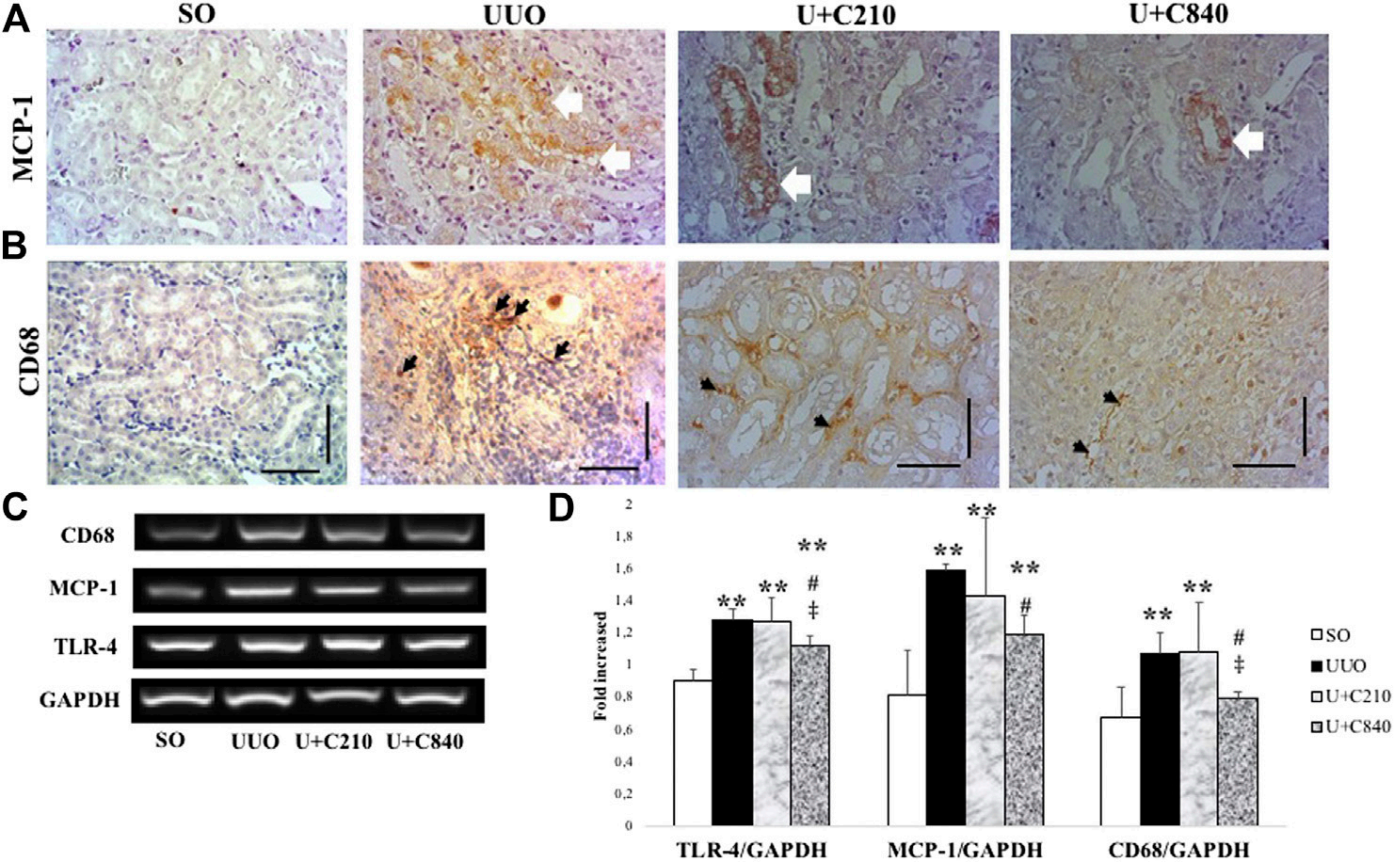
CeA treatment attenuated inflammation. **(A)** Immunostaining of MCP‐1 demonstrated no staining in SO group, however positive staining in epithelial cells and interstitial areas of kidney from UUO group (white arrows). **(B)** Histopathological appearance of each group shown in immunostaining for CD‐68. **(C,D)** Representative picture and semiquantitative analysis of electrophoresis results of RT‐PCR analysis showing CD‐68, MCP‐1, and TLR‐4 mRNA expressions. *p < 0.05 vs SO; **p < 0.01 VS SO, ***p < 0.001 vs SO, #p < 0.05 vs UUO; ##p < 0.01 vs UUO, ‡p < 0.05 vs U+C210, ‡‡p < 0.01 vs U+C210.

Immunostaining showed expression of MCP-1 in epithelial cells and interstitial areas of UUO group, which associated with macrophage infiltration (CD68 positive staining) (Figures 4A,B). CeA treated groups showed lower expression with downregulation of MCP-1 immunostaining and reduction of macrophage infiltration. Quantification of inflammation cascade using RT-PCR (Reverse transcriptase PCR) demonstrated significant higher mRNA expression of TLR-4, MCP-1 and CD68 in UUO group. The U+C840 group demonstrated attenuation of inflammation as shown by a significantly lower mRNA expression of TLR4, MCP-1, and CD68 compared to UUO (Figure 4).

## Discussion

Our study revealed that treatment with CeA extract attenuates progression of kidney fibrosis by reducing mesenchymal transition/myofibroblast formation, inflammation, and tubular injury. The UUO model induced monocyte activation and macrophage infiltration, produced angiotensin II, and stimulated the production of nuclear factor-κB, thus attracting more macrophages to the site of injury. Classically, activated monocytes transform into macrophages, produce cytokines such as TGF-β1, and cause fibrosis with increasing ECM deposition (Chevalier et al., 2009). TGF-β is a central factor in some conditions associated with progressive renal diseases and contributes to tubular loss; fibroblast recruitment, proliferation, and activation; myofibroblast formation; and ECM accumulation (Robson et al., 2006). Fibroblasts are activated mostly by tissue injury then undergoes myofibroblast transition (Figure 2), while the process of resident interstitial fibrosis is activated by some cytokines, such as TGF-β1, platelet-derived growth factor (PDGF), and fibroblast growth factor 2 (FGF-2) (Strutz and Zeisberg, 2006). Our results demonstrated reduction of fibrosis and myofibroblast formation with downregulation of TGF-β and α-SMA (myofibroblast marker) expression in the CeA-treated groups, especially in the U+C840 group (Figure 1), with the higher dose of CeA extract.

Xu et al. also demonstrated that high doses of CeA attenuated fibrosis with the inhibition of Smad-dependent TGF-β1 transduction signal, which was associated with decreased tubular injury and fibroblast proliferation, activation, and recruitment. However, treatment with a low dose of CeA (1 mg/kg BB) showed no improvement in fibrosis. Thus, it was concluded that CeA is effective in lowering renal interstitial fibrosis depending on the dose, specifically an intermediate to high dose regimen (Xu et al., 2013). TGF-β induces mesenchymal transition of many cells, not only fibroblast, but also other cells such as endothelial cells through endothelial–mesenchymal transition (EndMT) and epithelial cells through EMT (Chevalier et al., 2009). The Snail and FSP-1 protein genes have a role in promoting phenotype transformations, which is increased by TGF-β. Snail gene promotes the transformation of epithelial cells into mesenchymal cells, which is a crucial process in embryonic development and also plays a role in the acquisition of invasive and migratory properties during tumor progression (Nieto, 2002). The mRNA expression of Snail1 is enhanced in obstructive nephropathy compared with normal kidneys (Yoshino et al., 2007). In another study, EMT was induced within 24 h after UUO, along with upregulation of the mRNA expression of Snail1 in the kidney (Lange-Sperandio et al., 2007). According to Sato et al., Snail is expressed within 7 days of UUO in mice (Sato, et al., 2003). The results of our study revealed the attenuation of fibrosis associated with the downregulation of mRNA expression of Snail and FSP-1, as a part of downstream signaling of TGF-β.

We also demonstrated the reduction of E-cadherin, which plays a role in EMT, with the upregulation of vimentin in UUO. EMT can be described as the enhancement of Snail1 and vimentin expression and decrease in E-cadherin transcription (Xu et al., 2013). EMT, which is induced by Snail expression, promotes the repression of epithelial markers such as E-cadherin (Nieto, 2002). According to a study conducted by Yang and Liu, E-cadherin expression in the UUO model was lost in an early stage, at 3–7 days (Yang and Liu, 2001). Downregulation of E-cadherin expression and enhancement of α-SMA expression in the UUO model occurs within 7 days (Sato, et al., 2003). EMT induction causes epithelial cells to lose their basic characteristics and transform into mesenchymal cells, with the availability of vimentin and a flattening phenotype (Park et al., 2008). Thus, vimentin might be expressed because the epithelial cell loses its characteristics and is transformed into a mesenchymal cell (Park et al., 2008). In EMT, there is a cytoskeletal change that can be detected using the vimentin marker (Kriz et al., 2011). According to a study by Lange-Speradino, enhancement of vimentin expression as a mesenchymal marker is observed within 5 days after UUO (Lange-Sperandio et al., 2007). It seems CeA attenuates mesenchymal transition and interstitial cells expansion in kidney fibrosis. CeA is well-known for its various components, including asiatic acid (AA) (Maquart et al., 1990). AA is responsible for reducing renal tubulointerstitial fibrosis through inhibition of TGF-β expression (Xu et al., 2013) Moreover, our study demonstrated that the downregulation of TGF-β may affect the attenuation of EMT, with upregulation of E-cadherin and downregulation of vimentin, thus reducing tubular injury in mice treated with 840 mg/kg BW of CeA. The downregulation of TGF-β1 signaling associates with reduction of mesenchymal transition from fibroblast and epithelial cells. Elucidating and quantification of fibroblast expansion and myofibroblast formation or using genetic lineage study with CeA treatment may give better understanding for next research.

Inflammation also plays an important role in the development of kidney fibrosis, with activation of TLR4. TLR4 acts as the key component in inducing proinflammatory responses (Molteni et al., 2016) and promotes chemokine secretion, which contributes to the selective recruitment of monocytes, neutrophils, and lymphocytes. MCP-1 is a key regulator of chemokines and regulates monocyte/macrophage migration and infiltration (Deshmane et al., 2009). CeA potentially contributes to anti-inflammatory processes (Gohil et al., 2019), as shown in our study, with the reduction of mRNA expression of TLR4, MCP-1, and CD68. Attenuation of inflammation also occurred in an animal model of allergic dermatitis, in which the animal was administered a titrated dose of CeA extract (Park et al., 2017). Elucidating active compounds of CeA may give better understanding in the renoprotective effect of CeA. Pentacyclic triterpenoid is the most abundant terpenoid in CeA, which reflect quality biomarker of CeA (Zheng and Qin, 2007). Main derivate of pentacyclic triterpenoid in CeA are *asiatic acid (AA)*, *madecassic acid*, *madasiatic acid*, *asiaticoside* dan *madecassoside* (Orhan, 2012). According to Chen et al.*,* AA reduces the expression of various inflammatory factors such as lipopolysaccharides (Kriz et al., 2011). Treatment with AA, an active component of CeA, in a kidney fibrosis model also demonstrated a reduction in MCP-1 expression, which is associated with downregulation of TGF-β and α-SMA. A higher dose of AA resulted in better results in a previous study (Xu et al., 2013), similar to the results of our study and another *in vitro* study (Chippada et al., 2011). Next research for elucidating the active compounds for renoprotective may be performed for continuing this study.

## Conclusion

Treatment with *Centella asiatica* attenuated kidney fibrosis by reducing mesenchymal transition, collagen deposition, and inflammation, in a mouse model of kidney fibrosis.

## Data Availability

The original contributions presented in the study are included in the article/Supplementary Material, further inquiries can be directed to the corresponding author.
